# Dismissing Attachment Characteristics Dynamically Modulate Brain Networks Subserving Social Aversion

**DOI:** 10.3389/fnhum.2016.00077

**Published:** 2016-03-09

**Authors:** Anna Linda Krause, Viola Borchardt, Meng Li, Marie-José van Tol, Liliana Ramona Demenescu, Bernhard Strauss, Helmut Kirchmann, Anna Buchheim, Coraline D. Metzger, Tobias Nolte, Martin Walter

**Affiliations:** ^1^Clinical Affective Neuroimaging Laboratory, Otto von Guericke UniversityMagdeburg, Germany; ^2^Department of Psychiatry and Psychotherapy, Otto von Guericke UniversityMagdeburg, Germany; ^3^Leibniz Institute for NeurobiologyMagdeburg, Germany; ^4^Department of Neurology, Otto von Guericke University, MagdeburgGermany; ^5^University of Groningen, Neuroimaging Center, University Medical Center GroningenGroningen, Netherlands; ^6^Institute of Psychosocial Medicine and Psychotherapy, University Hospital JenaJena, Germany; ^7^Institute of Psychology, University of InnsbruckInnsbruck, Austria; ^8^Center for Behavioral Brain Sciences (CBBS)Magdeburg, Germany; ^9^Institute for Cognitive Neurology and Dementia Research (IKND)Magdeburg, Germany; ^10^German Center for Neurodegenerative Diseases (DZNE)Magdeburg, Germany; ^11^Anna Freud CentreLondon, UK; ^12^Wellcome Trust Centre for Neuroimaging, University College of LondonLondon, UK; ^13^Department of Psychiatry, Eberhard Karls UniversityTübingen, Germany

**Keywords:** functional connectivity, individual differences, attachment, cognitive schema, resting-state

## Abstract

Attachment patterns influence actions, thoughts and feeling through a person’s “inner working model”. Speech charged with attachment-dependent content was proposed to modulate the activation of cognitive-emotional schemata in listeners. We performed a 7 Tesla rest-task-rest functional magnetic resonance imaging (fMRI)-experiment, presenting auditory narratives prototypical of dismissing attachment representations to investigate their effect on 23 healthy males. We then examined effects of participants’ attachment style and childhood trauma on brain state changes using seed-based functional connectivity (FC) analyses, and finally tested whether subjective differences in responsivity to narratives could be predicted by baseline network states. In comparison to a baseline state, we observed increased FC in a previously described “social aversion network” including dorsal anterior cingulated cortex (dACC) and left anterior middle temporal gyrus (aMTG) specifically after exposure to insecure-dismissing attachment narratives. Increased dACC-seeded FC within the social aversion network was positively related to the participants’ avoidant attachment style and presence of a history of childhood trauma. Anxious attachment style on the other hand was positively correlated with FC between the dACC and a region outside of the “social aversion network”, namely the dorsolateral prefrontal cortex, which suggests decreased network segregation as a function of anxious attachment. Finally, the extent of subjective experience of friendliness towards the dismissing narrative was predicted by low baseline FC-values between hippocampus and inferior parietal lobule (IPL). Taken together, our study demonstrates an activation of networks related to social aversion in terms of increased connectivity after listening to insecure-dismissing attachment narratives. A causal interrelation of brain state changes and subsequent changes in social reactivity was further supported by our observation of direct prediction of neuronal responses by individual attachment and trauma characteristics and reversely prediction of subjective experience by intrinsic functional connections. We consider these findings of activation of within-network and between-network connectivity modulated by inter-individual differences as substantial for the understanding of interpersonal processes, particularly in clinical settings.

## Introduction

The perception of an interpersonal encounter and thus the brain’s dynamic response to it depends on many aspects, including the listener’s past experiences, their current mood, attitudes, attention and cognitive performance. The basis for certain patterns of interpersonal behavior is already formed in early childhood. Individuals begin to develop an “internal working model” (IWM) of relational expectations combined with a set of likely responses towards interactions with others. These may in part find their origin in experiences with caregivers in early childhood (Bowlby, 1969/1982, 1973, 1980, 1988). This individual IWM forms their attachment representation, that influences interpersonal behavior such as approach/avoidance towards another individual or friendly/hostile reactions towards someone else. Different childhood or life experiences therefore lead to discernible types of attachment representations and corroborating expressions in behavior and discourse (e.g., Cassidy, 2008).

According to this theory of attachment, “attachment representations” can be discerned into secure and different insecure types (Hesse, 2008). Interestingly, attachment representations are part of our communication style and it has been shown that secure and insecure attachment representations can be differentiated based on the coherence level of a person’s speech (Hesse, 2008). Typically, securely attached individuals report past experiences in a coherent manner whereas narratives of insecure individuals are characterized by an incoherent discourse. Broadly, insecure attachment types can be subdivided in insecure-dismissing (or avoidant) and insecure-preoccupied (or anxious) styles, according to classification by the adult attachment interview (AAI) or questionaires such as the Experience in Close Relationships Questionnaire (ECR; Ehrenthal et al., 2009). Individuals with an insecure-preoccupied attachment representation tend to have long conversational turns and focus on affective aspects of experiences such as helplessness and anger. In contrast, insecure-dismissing individuals, report fragmented or abstract recollections of memories with significant others, which may even contradict previous statements (George et al., 1985; Buchheim and Mergenthaler, 2000; Hesse, 2008).^1^ The specific discourse characteristic of attachment-representations is known to influence the counterpart, by activation of certain schemata underpinning interpersonal disposition. Schemata are patterns of thought and behavior including internal representations of oneself, others and the environment. Their activation, as explained in the cognitive model of depression (Beck, 1967), can be seen as a psychopathologically relevant “carry-over” effect of an environmental trigger. Moreover, once activated by a salient internal or external stimulus, schemata influence the subsequent processing of social information and for instance, compromise mentalization capacities (Nolte et al., 2013). Furthermore, they impact attention towards negative or positive stimuli, which may bias processing and memory formation of new incoming information (Disner et al., 2011).

Investigating the influence of attachment representations on subsequent mental states, Kirchmann et al. (2011) revealed carry-over effects of prototypical audio narratives of different attachment representations (secure, insecure-dismissing and insecure-preoccupied) on self-reported well-being and interpersonal dispositions of the listener conceptualized as countertransference (see below). These conscious and unconscious responses were not only influenced by the content of the narrative but, more importantly, also by the different characteristic speech patterns of the securely and insecurely individuals whose recorded narratives were used as stimuli (Martin et al., 2007).

The “classical” concept of countertransference was first defined by the psychoanalyst Freud as “…the patient’s influence on [the therapist’s] unconscious feelings…” (Freud, 1910/1957, p. 144) resulting from the patient’s influence on the therapist. Freud (1910/1957, 1912/1959) considered countertransference as an unrequested process, which is associated to unresolved conflicts of the clinician. However, subsequent authors emphasized the importance of countertransference reactions for the therapeutic process and broadened the definition to the therapist’s emotions towards the patient comprising, to differing degrees, also an objective aspect not depending on the therapist’s own intrapsychic conflicts, but instead a response which the patient evokes in others and is thus related to the patient’s behavior (e.g., Winnicott, 1949; Heimann, 1950; Kernberg, 1965; Gabbard, 2001). Generally, countertransference reactions can be seen as one’s feelings towards the counterpart.

In a review article, Vrtička and Vuilleumier (2012) emphasized that in a novel interpersonal situation the first mental reaction is an affective evaluation of the situation, which is underpinned by the recruitment of specific brain networks important for the processing of social context. The first rapid or automatic processing of such a situation includes the decision to approach or avoid a stimulus or person. Brain regions important for social approach comprise ventral tegmental area (VTA), pituitary/hypothalamus, striatum, and ventral medial orbitofrontal cortex (OFC). Regions associated with fear and negative affect such as amygdala, hippocampus, insula, anterior cingulate cortex (ACC), and anterior temporal pole (ATP) are activated by aversive social events (Vrtička and Vuilleumier, 2012). The activation of these regions varies with individual differences: individuals with an insecure-avoidant attachment, for example, showed decreased activation in dorsal ACC in situations of social exclusion (DeWall et al., 2012) as well as weaker responses to sad and fearful faces (Zhang et al., 2008; Suslow et al., 2009). An insecure-anxious attachment style, in contrast, has been associated with an increased activation of the “social aversion system” in response to negative social cues (e.g., Lemche et al., 2006; Vrtička et al., 2008; DeWall et al., 2012). Furthermore, suppression of resting state activity has been observed in affective, empathy and default mode brain networks following emotional movie clips, emotional biographic narratives or social stress respectively (Eryilmaz et al., 2011; Vaisvaser et al., 2013; Borchardt et al., 2015), suggesting that emotional or social stress “carry-over” effects are associated with temporary alterations of the brain’s dynamic baseline. Consequently, such a shift in baseline intrinsic connection would have an effect on individual responses towards explicit subsequent situations.

Whether speech patterns characteristic of insecure attachment style really have similar effects on brain dynamics during rest, which could explain reactive avoidant or approaching behavioral tendencies, has not been investigated so far.

For the current study, we hypothesized that exposure to discourse characterized by an avoidant attachment representation may similarly influence intrinsic brain dynamics during rest between brain regions important for avoidant or approaching behavior, in contrast to speech characterized by a secure attachment style. Taken together, with neurophysiological and psychological evidence for considerable carry-over effects, this may provide a neuronal trace of what is described as schema activation in cognitive behavioral psychotherapy (Disner et al., 2011). Such influence on subsequent behavior and psychological states is thought to be mediated by prolonged changes of the intrinsic functional network architecture, which can be investigated using resting-state functional magnetic resonance imaging (fMRI). Importantly, we hypothesize that such effects are subject to inter-individual variability which can be best explained by the individual’s own attachment style. Individuals with an insecure attachment style, characterized by attachment anxiety and avoidance are thought to be more susceptible to behavioral changes and resting-state connectivity changes in a network relevant to process experience of social aversion.

Therefore, we investigated for the first time effects of attachment on subsequent resting-state brain responsivity and their modulation by individuals’ attachment styles.

## Materials and Methods

### Participants

Twenty three healthy male, right-handed participants (mean age: 29.8 years, SD: 3.5 years) underwent fMRI.

All participants were screened for psychiatric, neurological or medical illness using the Mini-International Neuropsychiatric Interview (MINI), the Hamilton Depression Scale (HAM-D) and the Young Mania Rating Scale (YMRS) to ensure the absence of any psychiatric disorders (Sheehan et al., 1998) according to ICD-10 criteria. In addition to psychopathology, exclusion criteria included ineligibility to undergo MRI.

The study was approved by the institutional review board of the University of Magdeburg, Germany, and all participants provided written informed consent according to the Declaration of Helsinki.

### Stimuli for the fMRI Experiment

Every participant listened to three narratives, characteristic for secure, insecure-dismissing and insecure-preoccupied attachment representation. These narratives were based on the AAI (George et al., 1985). Questions of this semi-structured interview focus on relevant past attachment situations with caregivers, potential losses and traumata—mainly in early childhood. The used narratives are prototypical excerpts of question 3 and 4 from the AAI in which the interviewees are asked to describe characteristic relationship examples with their father and mother in childhood. They belong to the AAI categories dismissing (Ds1/2), preoccupied (E2) and secure-autonomous (F3). To avoid the impact of different vocal qualities and to ensure anonymity of the interviewees, participants listened to recordings of the transcripts recited by AB. These stimuli have been validated and utilized in prior research by Martin et al. (2007) and Kirchmann et al. (2011). In these studies, the length of the narratives differed largely. To avoid possible confounds due to different length of the original narratives, the two longer recordings were shortened to a duration of 4:58 min (insecure-preoccupied) and 4:08 min (secure-autonomous) to match the naturally shorter dismissing narrative (3:46 min). During the process of shortening of the audio stimuli, attention was paid to preserve attachment specific content and speech characteristics (which was cross-checked by a reliable and experienced AAI rater).

### Data Acquisition and Procedure

Ultra-high field fMRI was chosen for its capacity to measure high spatial resolutions, which are very beneficial for the investigation of subcortical nuclear structures, highly relevant for affective and social processing (Walter et al., 2008b; Metzger et al., 2013a).

Resting-state BOLD data were acquired on a 7 T whole body MR system (Siemens, Erlangen, Germany) with a 32-channel receiver coil, using an EPI sequence (TR 2.61 s, TE 22 ms, 240 time points, 50 slices, voxel size 1.6 mm isotropic, flip angle 90°). Reconstruction of functional imaging data included a distortion correction and an inbuilt online-motion correction (Zaitsev et al., 2004; Speck et al., 2008). Motion corrected during this procedure was recorded for further analysis. Additionally residual motion was estimated using DPARSFA V2.3 (Yan and Zang, 2010; Song et al., 2011) motion detection.

T1-weighted anatomical reference data were imaged using 3D-MPRAGE (1 mm isotropic resolution, TE 2.01 s, TI 1050 ms, TR 1700 ms, flip angle 5°). The participants were instructed to lay still inside the scanner and for the resting-state scans in specific with their eyes closed without thinking of anyhing particular but without falling asleep. Motion was minimized using soft pads fitted over the ears and participants were given ear-plugs to minimize noise.

**Figure 1:**
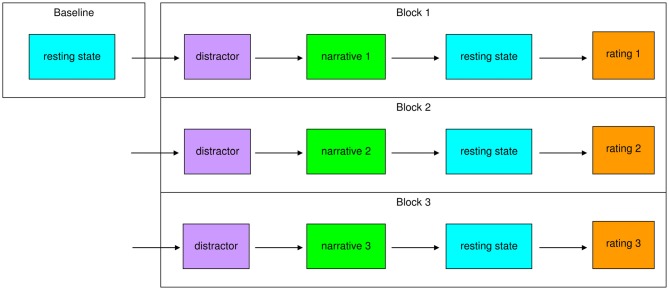
**Study design.** First, a 10 min baseline resting-state was measured. After a distractor (90 s), participants listened to the first narrative. Then a 10 min post-task resting-state was measured. Afterwards participants rated the narrative and their feelings. The block distractor–narrative–resting-state–rating was conducted for a second and third time, so every subject listened to all three narratives (secure, insecure-preoccupied, insecure-dismissing) in a randomized order over subjects.

We conducted a rest-task-rest design (Figure 1) with a 10 min baseline resting-state scan at the beginning. The aim of this design was to compare a baseline resting-state condition to a second resting-state scan, acquired directly after the short task of passive listening. A rest-task-rest design (Barnes et al., 2009) was used to provide the most naturalistic scenario for the presentation of the stimuli with the aim to elicit a natural response from the participants. The benefit for such a design lies in the free manipulation of brain activity during the actual stimulation, which avoids a necessity to arrange stimulation parallel to EPI noise, especially for longer auditory sequences. Secondly, the activation of schema-like processes might be more realistic if the actual social context is presented as naturalistic as possible, which is more difficult if repeated short sequences are required for an event related general linear model (GLM)-based analysis. Furthermore, we deemed this paradigm optimal to capture prolonged carry-over effects, which were thought to emerge after narrative exposure. Before each intervention, the participants had to complete a distractor task, which consisted of easy arithmetic problems (90 s) to ensure comparable baseline conditions. After this, they passively listened to the first narrative. Then we obtained 10 min post-task resting-state data. Afterwards, the participants were asked to rate their feelings and the narrative they had listened to with the questionnaires described below. This procedure was conducted three times so that every participant listened to all of the three narratives in one fMRI session. To control for order effects, the order of narrative presentation was randomized over participants.

### Questionnaires

The German version of the revised Experience in Close Relationships Questionnaire (ECR; Ehrenthal et al., 2009) was used to assess participants’ individual attachment style like avoidance and attachment anxiety. The Questionnaire contains 36 items estimating a persons’ needs, thoughts and behavior in relationships (e.g., “Just when my partner starts to get close to me I find myself pulling away.”) on a 7-point Likert scale (“not at all” to “exactly), including an attachment avoidance scale and an attachment anxiety scale.

As traumatic experiences in childhood play a substantial role in the development of different attachment styles, we utilized the German version of the Childhood Trauma Questionnaire (CTQ, Wingenfeld et al., 2010) to assess child maltreatment in our participants. The CTQ is a questionnaire with 28 items evaluating emotional, physical and sexual abuse and emotional and physical neglect on a 5-point Likert scale (“not at all” to “very often”).

Furthermore, we used a scale to assess countertransference reactions (one’s feelings towards the counterpart) of positive and negative quality based upon Mertens (2005) theoretical model of unconsciously elicited relational responses. This 16-item countertransference questionnaire was adapted from Martin et al. (2007). Sample items are: “Would you like to be a friend of this person?” or “Can you imagine how this person is feeling?” Participants had the possibility to rate with “rather yes”, “rather no” or “neither yes or no”, where high scores indicate a high tendency for social interaction.

To evaluate changes in participants’ wellbeing, we asked participants to complete the German Well-being scale (“Befindlichkeitsskala”, Bf-S’, Zerssen, 1976) at baseline and after every narrative. This well-established state-measurement starts with the sentence “At the moment I feel …” and consists of 28 pairs of extremes, e.g., “pessimistic” or “optimistic” or “neither nor”, where high scores indicate low well-being.

The Impact Message Inventory (IMI, Fingerle, 1998) is related to the interpersonal circumplex model and assesses someone’s reaction when being confronted with a person (“When I would be with this person, I would …”). In the current study the 8 item-subscale “friendly” was used. Participants evaluated their interpersonal reactions to the person listened to on a 4-point Likert scale (“not at all” to “exactly”). High scores indicate high estimation of friendliness.

The well-being scale was measured at baseline as well as after every narrative presentation. IMI and countertransference scale were measured after narrative presentation.

### Data Preprocessing and Functional Connectivity Analysis

Preprocessing was performed using SPM12 (Wellcome Trust Center for Neuroimaging, London, England; Friston et al., 1994), running under Matlab R2009a. Time series were slice-time acquisition corrected, realigned, coregistered to participant’s anatomical T1-weighted image (segmented and normalized into MNI space), normalized to MNI-space and smoothed (resampled to 2 mm cubic voxels) with a Gaussian kernel of 6 × 6 × 6 mm^3^ full-width-half-maximum. Since an online motion correction was applied, we requested residual head motion to be lower than 1.0 mm and 1.0 degree. One participant was excluded due to excessive movement, apparently uncontrollable by the correction algorithm. The instantaneous head displacement, also known as frame-wise displacement (FD), was calculated from derivatives of the six rigid-body realignment parameters estimated during standard volume realignment of the motion corrected data. Motion-induced artifacts were then minimized through censoring of motion confounded time points (i.e., “scrubbing”), presented by Power et al. (2012). The volume and its time-adjacent volume with larger head displacement (FD > 0.5 mm) were removed in the present study. After this, functional connectivity (FC) analysis was performed. This step was performed after preprocessing, which included deletion of the first 10 time points and temporal filtering (0.01–0.08 Hz) and regression of nuisance covariates (white matter, cerebrospinal fluid, motion and global signal) using DPARSFA V2.3 (Yan and Zang, 2010; Song et al., 2011).

Brain regions of the “social aversion network” (Vrtička and Vuilleumier, 2012) were selected as seed regions of interest (ROIs): amygdala, hippocampus and dorsal anterior cingulate cortex (dACC) of the baseline and dismissing condition. The ROI for hippocampus (*x* = −22, *y* = −20, *z* = −26, 8 mm sphere) has been described by Andrews-Hanna et al. (2010). The amygdala-ROI was based on the AAL atlas. For ACC-ROI, we used a modified version of the AAL atlas, containing a higher level of parcelling in cingulate cortex (Tzourio-Mazoyer et al., 2002; Yu et al., 2011; Lord et al., 2012).

Inter-individual differences: for every participant, correlation coefficients between the mean time course of each seed ROI and the time course of every voxel in the brain were calculated and then converted into three-dimensional zFC maps, using Fisher’s r-to-z transformation. Individuals’ zFC maps were entered into a paired *t*-test in SPM12 to determine the effect of narratives on seed-driven FC. We conducted a paired *t*-test to compare baseline to the narrative condition. A multiple regression analysis was performed to test for a correlation of post-narrative states with the individual scores of the questionnaires in the different scanning conditions for dACC, amygdala and hippocampus.

We further tested if subjective ratings of friendliness and countertransference following the dismissing narrative were also predictable from participants’ baseline resting state networks. This correlation analysis, in contrast to the above correlation of acute brain changes as a function of attachment trait markers, tested the inverse association of individual predisposition as represented in participants’ brain network states towards subsequent behavioral responsivity. We focused on subjective responses of friendliness after the dismissing state as it most strongly differed from secure conditions and the prediction of subsequent responsivity by baseline resting state connectivity followed a similar approach proposed by Metzger et al. (2013b) on participants’ behavioral reactivity towards pharmacological challenge. Again, a multiple regression analysis was performed to test for a correlation of post-narrative states with the individual scores of the questionnaires in the different scanning conditions for dACC, amygdala and hippocampus.

Changes from baseline were assessed using a paired *t*-test and multiple comparisons were corrected using false discovery rate (FDR, peak-level *p*-value = 0.05) on the whole brain level. The same correction was used for prediction of friendliness scores after the dismissing narrative by baseline connectivity within the “social aversion network”.

Statistical analyses were carried out using SPSS 20. A full factorial model was used to assess the effect of narrative type on individual wellbeing, friendliness and countertransference reactions. Correlation analyses were conducted for the scales of ECR and CTQ.

## Results

### Behavioral Effects of Narratives and Correlation of Attachment Style and Childhood Trauma

**Figure 2:**
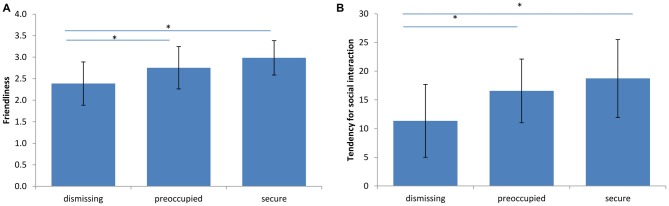
**Behavioral effects of narratives. (A)** Friendliness. A significant effect of narrative on countertransference reactions was observed (*n* = 23, *p < 0.03, *F*_(2,19)_ = 11.73). The dismissing narrative was rated as the least friendly. The bars represent the standard deviation between subjects. **(B)** Tendency for social interaction. A significant effect of narrative on countertransference reactions was observed (*n* = 23, *p < 0.004, *F*_(2,19)_ = 11.30). The participants showed the lowest tendency for potential social interaction with the dismissing narrative. The bars represent the standard deviation between participants.

A significant difference of evaluated friendliness was found between dismissing narratives when compared to preoccupied and secure ones (dismissing-preoccupied *p* = 0.022; dismissing-secure *p* < 0.001), but not between preoccupied and secure (preoccupied-secure *p* = 0.188; Figure 2A). This was paralleled by lowest tendency to engage in potential social interaction (measured with the countertransference-scale) with the dismissing prototype when compared to the preoccupied (*p* = 0.003) and to the secure narrative (*p* = 0.002; Figure 2B).

These specific reactions appeared in the absence of significant changes of wellbeing between conditions (dismissing-preoccupied *p* = 1.00, dismissing-secure *p* = 0.29, preoccupied-secure *p* = 1.00).

**Figure 3:**
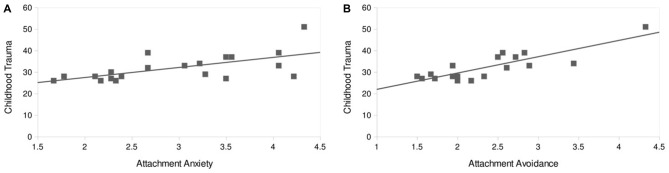
**Correlation of attachment characteristics with childhood trauma score.** Childhood Trauma Questionnaire (CTQ) score correlates positively with attachment anxiety (*r* = 0.66, *p* < 0.002; **A**) and attachment avoidance (*r* = 0.59, *p* < 0.006; **B**).

A significant positive correlation was found for the sum score of the CTQ with attachment anxiety (*r* = 0.66, *p* < 0.002) and attachment avoidance (*r* = 0.59, *p* < 0.006; Figure 3). Regarding the CTQ subscales, emotional abuse and emotional neglect both correlated positively with attachment anxiety (emotional abuse—attachment anxiety: *r* = 0.49, *p* = 0.026; emotional neglect—attachment anxiety: *r* = 0.44, *p* = 0.046) and attachment avoidance (emotional abuse—attachment avoidance: *r* = 0.59, *p* = 0.005; emotional neglect—attachment avoidance: *r* = 0.62, *p* = 0.003). As the subscales physical abuse, sexual abuse and physical neglect were not normally distributed, we conducted for theses scales the non-parametric Spearman’s rho—test. Physical abuse and physical neglect showed a positive correlation with attachment anxiety (physical abuse—attachment avoidance: *r* = 0.61, *p* = 0.003; physical neglect—attachment avoidance: *r* = 0.60, *p* = 0.005).

We did not find any significant correlations of HAM-D and YMRS with the CTQ total score and its subscores (HAMD-CTQ totalscore: *r* = 0.18, *p* = 0.434; YMRS-CTQ totalscore: *r* = −0.25, *p* = 0.283).

For descriptive statistics see Table 1.

**Table 1:** **Descriptive statistics**.

		Mean	Std	95% Confidence interval
Wellbeing (Bf-S)	Dismissing	36.74	11.15	31.91–41.56
	Preoccupied	35.10	7.98	31.65–38.56
	Secure	33.87	9.14	29.92–37.82
IMI (friendly)	Dismissing	2.39	0.50	2.17–2.60
	Preoccupied	2.75	0.49	2.54–3.00
	Secure	2.98	0.40	2.81–3.16
Counter-transference	Dismissing	11.35	6.34	8.61–14.09
	Preoccupied	16.57	5.54	14.17–18.97
	Secure	18.74	6.76	15.81–21.66
Childhood Trauma Questionnaire	Sum score	32.38	6.51	29.42–35.34
	Emotional abuse	6.38	1.53	5.68–7.08
	Physical abuse	5.71	1.49	5.04–6.39
	Sexual abuse	5.33	1.53	4.64–6.03
	Emotional neglect	8.10	2.39	7.01–9.18
	Physical neglect	6.19	1.94	5.31–7.07
Attachment Anxiety (ECR)		2.93	0.85	2.57–3.30
Attachment Avoidance (ECR)		2.31	0.70	2.01–2.61
Hamilton Depression Scale (Ham-D)		0.74	1.20	0.22–1.26
Young Mania Rating Scale		0.48	1.04	0.03–0.93

### fMRI Results

#### Main Effects of the Dismissing Narrative

**Figure 4:**
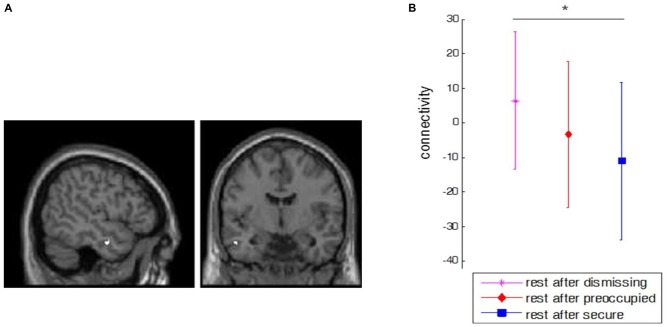
**Main effect of dismissing narrative. (A)** Depicted is the increase in functional connectivity (FC) between left dorsal anterior cingulated cortex (dACC; seed region) and left anterior middle temporal gyrus (aMTG; *x* = −50, *y* = −6, *z* = −24) after the dismissing narrative compared to baseline (*p* < 0.05, corrected, *Z* = 5.1, *k* = 8). The same peak coordinates were found for right dACC (*p* < 0.05, corrected, *Z* = 4.9, *k* = 6). **(B)** Comparison of FC between the seed dACC and anterior MTG between all narrative conditions. The bars represent the standard deviation between subjects. The black star indicates a significant difference (*p* < 0.01) between dismissing and secure conditions.

Changes in FC of the “social aversion network” were found when comparing between baseline and exposure to the dismissing narrative, but not in response to the other two narratives:

A stronger FC between dACC was found towards another component of the “social aversion network”, namely the left anterior middle temporal gyrus (aMTG; *x* = −50, *y* = −6, *z* = −24, left dACC: *Z* = 5.1, *k* = 8, *p* < 0.05, corrected; right dACC: *Z* = 4.9, *k* = 6, *p* < 0.05, corrected) after the dismissing condition compared to baseline resting-state (Figure 4A). A direct comparison of FC between dACC and anterior MTG between all conditions further revealed that this FC was significantly increased for dismissing against secure narrative conditions but not between secure and preoccupied (Figure 4B).

We found no significant changes of FC for amygdala or hippocampus.

#### Inter-Individual Differences

FC of dACC was then investigated to mediate effects of behavioral variability across participants related to attachment characteristics.

**Figure 5:**
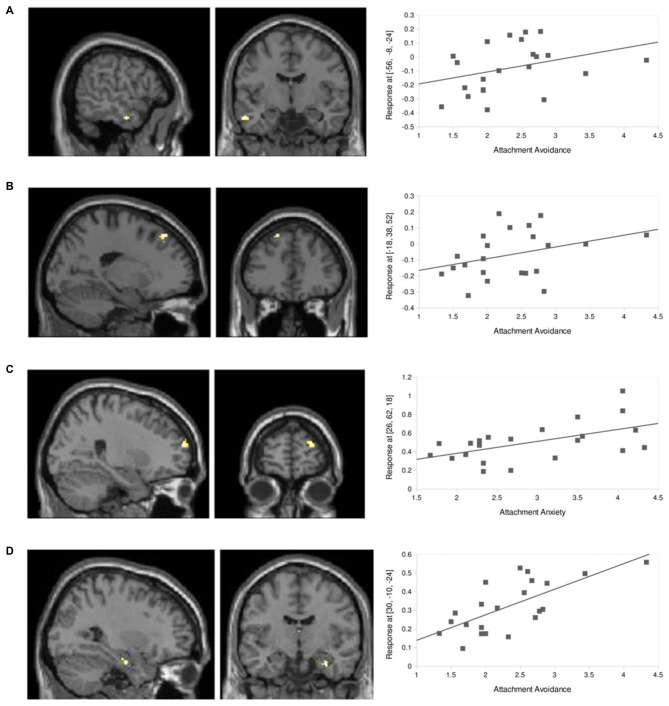
**Inter-individual differences. (A)** FC between right dACC and aMTG (*x* = −56, *y* = −8, *z* = −24) after the dismissing narrative showed a positive correlation with attachment avoidance (*Z* = 3.7, *k* = 18, *p* < 0.001, uncorrected, *r* = 0.34). **(B)** FC between right dACC and MPFC (*x* = −18, *y* = 38, *z* = 52) after the dismissing narrative showed a positive correlation with attachment avoidance (*Z* = 3.8, *k* = 21, *p* < 0.001, uncorrected, *r* = 0.34). **(C)** FC between left dACC and right DLPFC (*x* = 26, *y* = 62, *z* = 18) after the dismissing narrative showed a positive correlation with attachment anxiety (*p* < 0.001, uncorrected, *Z* = 4.1, *k* = 40, *r* = 0.54). **(D)** FC between right dACC and right hippocampus (*x* = 30, *y* = −10, *z* = −24) after the dismissing narrative showed a positive correlation with attachment avoidance (*p* < 0.001, uncorrected, *Z* = 3.6, *k* = 12, *r* = 0.69).

The main effect of dismissing narratives, FC between dACC and aMTG was further specifically correlated with attachment avoidance (*r* = 0.34), also when compared to effects of attachment anxiety (*x* = −56, *y* = −8, *z* = −24; *Z* = 3.7, *k* = 18, *p* < 0.001, uncorrected; Figure 5A). This was also the case for FC between dACC and left medial prefrontal cortex (MPFC; *x* = −18, *y* = 38, *z* = 52; *Z* = 3.8, *k* = 21, *p* < 0.001, uncorrected, *r* = 0.34; Figure 5B).

Inversely, FC between left dACC and right DLPFC specifically correlated negatively with attachment anxiety (*r* = 0.54) when compared to attachment avoidance (*x* = 26, *y* = 62, *z* = 18; *Z* = 4.1, *k* = 40, *p* < 0.001, uncorrected; Figure 5C).

In contrast, FC between right dACC and right hippocampus (*x* = 30, *y* = −10, *z* = −24) after the dismissing narrative correlated positively (*r* = 0.69) with attachment avoidance scores (*Z* = 3.6, *k* = 12, *p* < 0.001, uncorrected; Figure 5D), however this effect was not revealed specific by direct comparison with attachment anxiety.

#### Childhood Trauma Related Influences on Regions Involved in Social Aversion

**Figure 6:**
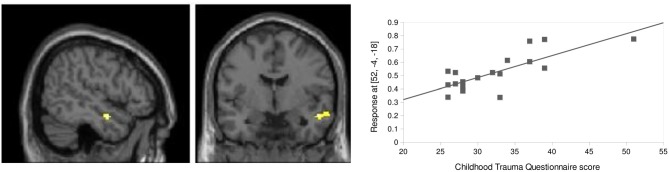
**Childhood trauma related influences.** FC between right amygdala and right aMTG (*x* = 52, *y* = −4, *z* = −18) at baseline showed a positive correlation with CTQ total score (*r* = 0.79, *p* = 0.008, *k* = 43, small volume corrected for an AAL-derived MTG ROI on a cluster level).

Baseline FC of aMTG, as a target region for amygdala seeded FC, was positively correlated with participants (*n* = 20 due to missing CTQ data) CTQ total score (*p* = 0.008, *k* = 43, small volume corrected for an AAL-derived MTG ROI; Figure 6). To gain a deeper insight into the effect of the subscales of the CTQ, we conducted multiple regression analyses with the same ROI and the scores of the CTQ subscales. The subscale physical neglect is driving the effect (*p* < 0.001, *k* = 102, small volume corrected for an AAL-derived MTG ROI) followed by emotional neglect (*p* = 0.010, *k* = 39, small volume corrected for an AAL-derived MTG ROI) and emotional abuse (*p* = 0.048, *k* = 21, small volume corrected for an AAL-derived MTG ROI).

#### Baseline Network Prediction of Subjective Response

**Figure 7:**
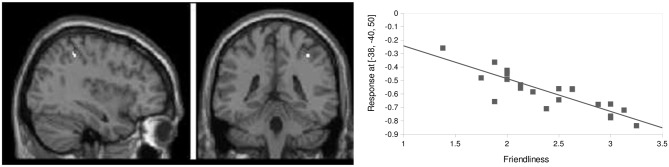
**Baseline prediction by friendliness scores.** FC between left hippocampus and left inferior parietal lobule (IPL; *x* = 38, *y* = −40, *z* = 50) correlated negatively with friendliness ratings (*p* < 0.05, corrected, *Z* = 5.17, *k* = 9, *r* = −0.86).

In a *post hoc* analysis, the general predisposition to experiencing aversion following dismissing narrative was further examined using baseline resting-state measures. An exploratory multiple regression analysis with friendliness and countertransference scores recorded for the dismissing condition as a predictor was performed for baseline resting-state connectivity of dACC, amygdala and hippocampus. A significant negative correlation (*p* < 0.05, corrected, *Z* = 5.17, *k* = 9, *r* = −0.86) was found for FC between left hippocampus and right inferior parietal lobule (IPL; *x* = 38, *y* = −40, *z* = 50) with subjective friendliness ratings. Participants with high FC between these two regions at baseline reported low subjective friendliness ratings of the subsequent dismissing narrative (Figure 7).

For the countertransference scores, we found no significant correlations.

## Discussion

We demonstrated that FC in a “social aversion network” was increased specifically after presentation of the prototypical narrative of a person with insecure-dismissing attachment representation. This key finding mirrors the behavioral countertransference reactions of listeners towards insecure-dismissing attachment characterized by the lowest rating of friendliness and tendency for social interaction (countertransference-reactions)—in comparison to the secure and the insecure-preoccupied narrative.

Particularly, the connectivity of the dACC with the hippocampus as well as the aMTG and MPFC showed a positive correlation with participants’ own individual attachment *avoidance* levels. Furthermore, participants’ attachment *anxiety* was correlated with dACC connectivity to the DLPFC which is outside the postulated “social aversion network”. In the same direction the participants’ general susceptibility towards dismissing content as expressed by subjective evaluations of *friendliness* was correlated with FC between another major region of the “social aversion network”, the hippocampus, and the MPFC and parietal cortex, which are also not part of the “social aversion network”. Together, these findings support the hypothesis that listening to dismissing attachment narratives leads to a specific increase of FC within the “social aversion network”—while interactions of these nodes with regions outside this network, such as DLPFC and parietal cortex are mediated especially by the listener’s attachment *anxiety* and evaluation of *friendliness*.

### Activation of the “Social Aversion Network”

Our findings are in line with the interpretation that listening particularly to the dismissing narrative triggers a prolonged activation of the “social aversion network” in healthy participants related to a schema activation as described in depressed patients (Disner et al., 2011). This set of interconnected brain regions directly relates to self-reported avoidance and to anxiety and friendliness indices in the current sample via the recruitment of additional brain regions. This interpretation is furthermore supported by the corroborating behavioral results which highlight a reactivity pattern indicative of “disengagement” from someone else’s narrative when characteristic of a dismissing attachment representation.

Recent neuroimaging findings suggest that the dACC plays a key role in processing social aversion, e.g., social exclusion induced by the cyberball paradigm (DeWall et al., 2012). Furthermore, Buchheim et al. (2008) reported an increased activity in the dACC in response to talking to attachment-related pictures representing loneliness in patients with borderline personality disorder who are characterized by a predominance of insecure unresolved attachment representations with respect to attachment trauma. Gillath et al. (2005) found increased activity in ATP, hippocampus and dACC of anxiously attached participants when thinking about negative emotions. While activity in OFC was decreased when suppressing these negative thoughts, activity in ATP and OFC was even inversely correlated (Gillath et al., 2005). Moreover, ATP was found to be an important neural correlate of sadness and grief states (Lévesque et al., 2003; Kersting et al., 2009). Vrtička and Vuilleumier (2012) emphasized not only the important role of the “social aversion network” for the processing of social context, but also that of regions essential for social approach and for more controlled cognitive reappraisal processes such as the prefrontal cortex (Vrtička and Vuilleumier, 2012).

We found that these important regions within the “social aversion network”, dACC and aMTG, show a higher FC after listening to the dismissing narrative implying a detachment of speech patterns characteristic for dismissing attachment representation.

### Inter-Individual Differences

Attachment avoidance correlated with the FC between dACC and aMTG after the dismissing narrative. This correlation was specific for the listener’s own attachment avoidance as compared to attachment anxiety. This supports the activation or increased intrinsic connectivity within the “social aversion network” as a function of sensitivity to dismissing content.

A positive correlation of FC between dACC and hippocampus, another constituent of the “social aversion network”, was observed for resting state conditions following the dismissing narrative, however, this correlation could not be rendered significantly different when corrected for attachment anxiety. Such a distinct correlation for attachment avoidance was further the case for FC between dACC and MPFC after the dismissing narrative, however this was not predicted by our hypothesis given that MPFC had been assigned to a network subserving mental state representation. There is previous evidence for co-activation of ACC and MPFC during thought suppression in attachment avoidant individuals (Gillath et al., 2005). Gillath et al. (2005) also found ATP, dACC and hippocampus to be correlated with attachment anxiety, while we could not reproduce this finding.

We did however find specific effects for attachment anxiety, also when directly comparing with correlations of attachment avoidance: attachment anxiety influenced the change of connectivity in reaction to the preceding dismissing narrative between the “social aversion network” and dorsolateral prefrontal cortex. Of note, no effect of attachment anxiety on connectivity with other regions associated with amygdala processing such as the ventrolateral prefrontal cortex was found. Whether the DLPFC indeed plays a mediating role between social aversion and other crucial social-cognitive processes, such as for instance monitoring social processes, may remain a speculative interpretation at least on the basis of our data. The comparably low level of social threat experiences after the narrative may have led to an increased activation of neural structures underpinning successful reappraisal of potentially distressing information in anxious individuals rather than to an active induction of anxiety and thus changes of amygdala connectivity. Recently, Silvers et al. (2014) found that high intensity threats lead to stronger DLPFC activation compared to those of lower intensity. Similarly, individuals with high attachment anxiety may have interpreted the dismissing narrative as more intense and thus reacted with increased, yet still effective, reappraisal strategies (Silvers et al., 2014).

Following Ochsner et al. (2012) correlations after dismissing narratives with ventrolateral and DLPFC regions suggest an increased call for regions associated with explicit regulation of affective states in order to keep homeostasis or a healthy state, which is especially relevant since we measured healthy partipants. Although the direct function cannot be inferred from resting state network connectivity, this suggests that participants who currently show higher levels of attachment anxiety specifically need more regulatory resources to maintain homeostasis following dismissing narratives.

While DLPFC activation was related to threat (Silvers et al., 2014), activation of the ventral lateral prefrontal cortex during social feedback processing was found to correlate with attachment anxiety (Vrtička et al., 2014). To our knowledge, literature on DLPFC, especially in its modulation of connectivity with dACC during attachment processing is missing. Therefore, more work will be needed to explore this particular finding in the future.

### Childhood Trauma Related Influences on Regions Involved in Social Aversion

As experiences in childhood play an important role in the development of attachment style and childhood maltreatment is associated with an insecure attachment style (e.g., Cassidy, 2008), we expected CTQ scores to correlate with attachment avoidance and attachment anxiety scores measured with the ECR.

Interestingly, CTQ scores correlated with FC between regions within the “social aversion network”, namely amygdala and aMTG, at baseline. It is well known, that the amygdala is involved in the processing of fear and stress-related experiences (e.g., Keifer et al., 2015). Marusak et al. (2015) for example could show a “higher conflict-related amygdala reactivity” whereas in a more specific attachment context, Lemche et al. (2006) found an increased amygdala response to negative sentences with attachment content, which was also correlated with attachment insecurity. Examining intra- and extra-amygdaloid paths in healthy participants with and without early life stress (using the CTQ as a measure), Grant et al. (2015) found that participants with early life stress showed very complex and “atypical” amygdaloid connectivity compared to participants without early life stress. Furthermore, individuals with a history of childhood maltreatment (measured with the CTQ) were found to show a higher amygdala responsiveness to threat-related facial expressions, which is underlying one aspect of vulnerability for affective disorders and post-traumatic stress disorder (Dannlowski et al., 2012). These findings underpin the relevance of the amygdala and its connections to the “social aversion network” in the context of childhood trauma, attachment style and psychiatric disorders. In general, disturbances in network reactivity related to childhood trauma, which might result for instance in a higher activity of the “social aversion network”, seem likely as childhood trauma results in disturbances of brain development and traumatic experiences are consequently incorporated in brain connectivity (Glaser, 2014).

Regarding the CTQ subscales, in our sample the subscale physical neglect is driving the effect followed by emotional neglect and emotional abuse in contrast to other studies where the emotional subscales often showed the strongest effects (e.g., Dannlowski et al., 2012).

In the current study, we found a correlation of aMTG in its FC (after dismissing states) to dACC with attachment avoidance and further a correlation of aMTG FC (at baseline) to amygdala with CTQ. We further observed correlations between attachment avoidance and CTQ. Further studies should thus try to directly address this complex interrelation and potential underlying circuits by focusing more directly on this task-rest relationship.

### Baseline Network Prediction of Subjective Response

In addition to changes in the “social aversion network” following the dismissing narrative and the accompanying inter-individual differences in attachment avoidance, we also observed a relevance of connectivity of a constituent of the “aversion network”, namely hippocampus, for individual predisposition to react to the dismissing narrative. Resting-state FC between hippocampus and IPL at baseline was negatively correlated with the friendliness perception regarding the speaker in the dismissing narrative (note that all three narratives were conveyed by the same person, thus differences in perception are due to content but not the speaker). The importance of the structures involved here would be in line with previous associations of subjective reactivity towards stressful social situations. Furthermore, Hertzman and Boyce (2010) or Letourneau et al. (2014) for example describe long-term-changes in the hypothalamic–pituitary–adrenal (HPA) system, the main component of stress response, related to the quality of early caregiving. This is an evidence for brain and body changes in individuals with a history of neglect or maltreatment, as often seen in insecure and disorganized individuals. As the hippocampus is strongly connected to the hypothalamus-pituitary-adrenal-axis it plays an important role in the evaluation of stressful social encounters (Foley and Kirschbaum, 2010) while the IPL is essential in social perception processes, e.g., in the distinction between self and other (Ruby and Decety, 2004; Lawrence et al., 2006). The association of their interconnectivity, especially as a baseline feature characterizing participants with stronger responsiveness to dismissing attachment information is novel and suggests further investigations of the link between IWMs of attachment and social stress systems.

### Clinical Implications

By means of understanding the neural correlates and etiological factors of affective disorders as well as different processes and impacts on outcome of psychotherapy, attachment characteristics, childhood trauma and countertransference reactions are meaningful aspects to be considered. According to Beck’s cognitive model of depression (Beck, 1967), depressive symptoms are induced and maintained when certain environmental triggers activate specific schemata stored in a person with an individual vulnerability. Significant factors of vulnerability are childhood maltreatment and attachment characteristics (e.g., Gilbert et al., 2009; Strauss, 2011). For the latter, a moderate increase of attachment security was found after psychotherapy (Kirchmann et al., 2012). In our study, we found carry-over effects similar to schema activation and countertransference reactions induced by narratives characteristic for attachment representations. Schema activation is not only fundamental for the understanding of depression (Clark et al., 1999), but attachment-related schemata are also expected to appear in the therapeutic alliance and influence therapy outcome substantially (e.g., Blatt et al., 2008; Muller, 2010; Schauenburg et al., 2010). The activation of the “social aversion network” after the dismissing narrative therefore is an important insight and needs to be considered especially in therapeutic relationships as the awareness of these mechanisms can be beneficial for the process of psychotherapy.

Brain networks involved in affective and anxiety disorders include for example ACC, hippocampus and amygdala (e.g., Disner et al., 2011; Nolte et al., 2011; Grotegerd et al., 2014). The understanding of these brain networks can be a clinically helpful step towards neuroimaging markers assessing the individual vulnerability for affective disorders.

### Limitations

Some limitations need to be considered. Firstly, while the sample size is quite large compared to other group analysis performed at high magnetic fields so far (Metzger et al., 2010) it is relatively small and suggests a cautious interpretation of the results. Secondly, all participants were young (mean age: 29.8 years) males. We chose to include only males to avoid sex differences and hormonal variations. At this point sex differences in attachment contexts are not fully understood and therefore we wanted to avoid this complicating factor. However, Ehrenthal et al. (2009) reported no differences in gender regarding levels of attachment anxiety and avoidance for the German version of the revised ECR. Nevertheless, the results cannot be generalized to females. Thirdly, although there was an attachment-related effect of the stimuli shown in prior research (Martin et al., 2007; Kirchmann et al., 2011) on a behavioral level, an additional effect of emotional arousal induced by the content of the stimuli cannot be excluded and needs to be considered when interpreting the results.

Furthermore, high field investigations are of particular danger to incomplete brain coverage due to signal losses. We therefore ensured that all regions of the “social aversion network” were covered. The small voxel sizes chosen here further add to signal rescue due to minimizing intra-voxel dephazing (Walter et al., 2008b). Nevertheless, some parts of hypothalamic areas were not covered in all participants. Given that this region is crucially involved in processing social interactions (Walter et al., 2008a), at least partial involvement of this region cannot be fully excluded. Higher spatial resolution would in part have resolved the issue of signal losses in hypothalamus (Metzger et al., 2010) but would have led to either restricted coverages or largely increased repetition times. New methods such as multiband EPI (Feinberg and Setsompop, 2013) may solve this issue for future studies.

## Conclusion

To conclude, this study revealed prolonged changes in brain activity in a network processing social aversion. This network was particularly activated after stimulation with dismissing attachment content and therefore may be seen as a representation of the neuronal correlates of schema activation—reflecting previous findings on a behavioral level (Martin et al., 2007; Kirchmann et al., 2011). Importantly, this activation of within-network and between-network connections was modulated by inter-individual differences of self-reported attachment avoidance and attachment anxiety respectively. To our knowledge, our study is the first to establish the effect of complex emotional inductions related to characteristic attachment speech patterns on specific and temporally robust FC changes. These findings have implications for the understanding of interpersonal processes, particularly in clinical settings, especially the process of psychotherapy, where an activation of attachment-related schemata of both patient and clinician are likely to occur (e.g., Blatt et al., 2008; Muller, 2010; Schauenburg et al., 2010).

## Author Contributions

ALK, BS, HK, AB, CDM, TN and MW did substantial work on the design of the study. AB, BS and HK provided the narratives and previous experiences with the design. ALK and MW were responsible for the data acquisition. ALK, VB, ML, M-JvT, LRD, TN and MW contributed to data anlaysis and interpretation of the data. ALK, VB, M-JvT, TN and MW drafted the work and all co-authors were revising and improving it critically. All co-authors approved the final version and agree to be accountable for all aspects of the work.

## Funding

This work was supported by DFG-SFB 779, the DAAD and a scholarship by the Otto v. Guericke University to ALK. M-JvT was supported by a VENI grant (NWO grant number 016.156.077).

## Conflict of Interest Statement

The authors declare that the research was conducted in the absence of any commercial or financial relationships that could be construed as a potential conflict of interest.

## Footnotes

^1^It must be pointed out that the attachment terminology is quite complex. Talking about attachment *representation* (e.g., assessed by the AAI) the terms “(insecure-) dismissing” for individuals scoring high on avoidance scales and “(insecure-) preoccupied” for individuals scoring high on anxiety scales or preoccupation with anger are used. Talking about the attachment *style* (and therefore self-reported aspects) the terms “(insecure-) avoidant” and “(insecure-) anxious” are used.
